# Geotechnical and Shear Behavior of Novel Lunar Regolith Simulants TUBS-M, TUBS-T, and TUBS-I

**DOI:** 10.3390/ma15238561

**Published:** 2022-12-01

**Authors:** Lisa Windisch, Stefan Linke, Magnus Jütte, Julian Baasch, Arno Kwade, Enrico Stoll, Carsten Schilde

**Affiliations:** 1 Institute for Particle Technology, TU Braunschweig, Volkmaroder Str. 5, 38104 Braunschweig, Germany; 2 Chair of Space Technology, TU Berlin, Marchstr. 12-14, 10587 Berlin, Germany

**Keywords:** lunar soil, lunar regolith simulant, in situ resource utilization, ISRU, shear test, lunar exploration, geotechnical properties, lunar dust

## Abstract

The return to the Moon is an important short-term goal of NASA and other international space agencies. To minimize mission risks, technologies, such as rovers or regolith processing systems, must be developed and tested on Earth using lunar regolith simulants that closely resemble the properties of real lunar soil. So far, no singular lunar simulant can cover the multitude of use cases that lunar regolith involves, and most available materials are poorly characterized. To overcome this major gap, a unique modular system for flexible adaptable novel lunar regolith simulants was developed and chemically characterized in earlier works. To supplement this, the present study provides comprehensive investigations regarding geotechnical properties of the three base regolith simulant systems: TUBS-M, TUBS-T, and TUBS-I. To evaluate the engineering and flow properties of these heterogeneous materials under various conditions, shear tests, particle size analyses, scanning electron microscope observations, and density investigations were conducted. It was shown that small grains <25 µm (lunar dust) are highly compressive and cohesive even at low external stress. They are particularly important as a large amount of fine dust is present in lunar regolith and simulants (*x*_50_ = 76.7 to 96.0 µm). Further, ring shear and densification tests revealed correlations with damage mechanisms caused by local stress peaks for grains in the mm range. In addition, an explanation for the occurrence of considerable differences in the literature-based data for particle sizes was established by comparing various measurement procedures. The present study shows detailed geotechnical investigations of novel lunar regolith simulants, which can be used for the development of equipment for future lunar exploration missions and in situ resource utilization under realistic conditions. The results also provide evidence about possible correlations and causes of known soil-induced mission risks that so far have mostly been described phenomenologically.

## 1. Introduction

### 1.1. Space Exploration and In Situ Resource Utilization

Driven by new technologies, a major boost to innovation is currently providing a significant expansion of the medium-term and long-term possibilities on the Moon and in space in general. However, in order to make lunar-based projects economically viable and sustainable, it is necessary to reduce the transportation needs of supplies and equipment from Earth to a minimum [1,2]. This makes the utilization of local in situ resources (ISRU) like lunar soil a key part to achieve these goals [3,4,5]. It significantly reduces the number of launches, cost, and risk [6,7]. In principle, it is possible to produce all structures needed for a lunar outpost from the material that covers the entire lunar surface [8,9,10].

ISRU involves any hardware or operation that utilizes and harnesses local resources in order to obtain products and services for space exploration. Traditional resources include e.g., soil, metals, alloys, sunlight, solar wind volatiles, or atmospheric gases. Robotic and human exploration also result in non-traditional resources like trash and wastes or residuals and spent landers [11].

In state-of-the-art approaches, there are three partial aspects that are considered mandatory for a sustainable plan for long-duration lunar habitats [12]: resource extraction from regolith, 3D manufacturing with local resources, and the recycling of disused materials. The first ISRU activities addressed the chemical extraction of oxygen and water from lunar regolith to produce breathable air, propellant, or human sustenance [13,14,15,16,17,18,19]. Additive in-space manufacturing processes are intended to mainly utilize local resources but also parts of disused spacecrafts. This goes hand in hand with the fact that several unmanned missions to the Moon are necessary before a fully manned station can be put into operation, and each mission leaves behind an unused landing structure. This complex sustainable material concept allows an almost completely closed material cycle [12]. ISRU research is furthermore concerned with the cultivation of plants, preferably in native soils [20], and the generation of critical consumables like propellants, fuel-cell reactants, and life-support consumables [21]. As a foundation for almost any infrastructure, the energy supply, e.g., by solar thermal power [22,23,24], must be ensured. At any point, the material covering the lunar (or any other celestial body’s) surface and its properties play a key role [25,26].

### 1.2. The Lunar Surface

The comprehension of the resources available on the Moon’s surface is crucial in order to develop appropriate methods and technologies for lunar missions. The lunar surface is covered with a loose, heterogeneous material, the so-called regolith (Greek for blanket of rocks), which overlays the unweathered solid rocks of the lunar crustal bedrock. This upper layer consists of small-grained rocks and dust and is sourced from the coarser bedrock underneath it. Most of the regolith material is a result of space weathering processes, such as the impacts of macro and micro meteroids and thermal weathering, as well as bombardment by charged atomic particles from the Sun [27]. These effects not only grind the coarser stones but also lead to the formation of new, extraterrestrial components like agglutinates. Five basic particle types make up the lunar soil [28]: mineral fragments, pristine crystalline rock fragments, breccia fragments, glasses of various kinds, and agglutinates [29]. Breccias and agglutinates are rock composites formed by meteorite impacts that (partially) melt the lunar bedrock, whereby particles are joined together. While breccias are formed by fragments of the bedrock, agglutinates are mainly composed of fine regolith particles, resulting in very diverse geometries, compositions, and hence, properties [28,30]. Glass particles, on the other hand, are formed from a complete melt and can vary greatly in their chemical composition depending on the original material [28,31]. A large proportion of glass particles—especially spherical ones—originate from volcanic activity. These various components lead to large local variations in the bulk and the geotechnical properties of the lunar regolith.

Lunar regolith in general is predominantly composed of two lithic minerals: anorthite and basalt. Anorthite is a Ca-rich plagioclase and represents the bright highland areas (i.e., terrae), whereas basalt is a mixture of anorthite feldspar and clinopyroxene, which is mainly found in the dark lowland areas (i.e., mare) [2,28]. However, the majority of the surface is very heterogeneous and consists of a diverse mixture of these two minerals, as well as the different thermally-altered particle types with various proportions and compositions. The wide range of equipment and technologies needed for settlement-related and exploration activities like habitat construction, spacecraft landing, and maneuvering or chemical extraction must be developed, designed, and tested for functionality as accurately as possible on Earth. In the case of the above-mentioned applications and considering the diverse composition of the lunar surface, the technologies must be able to work on or with cohesive, fine-grained rock material whose properties strongly fluctuate from one region to another. A total of nearly 382 kg lunar regolith was brought back over the course of six moon landings, resulting in nowhere near enough material to carry out crucial engineering studies [32]. In order to provide plenty material for experimental research as well as to create enough data to have a solid basis for the development of reality-based simulations and models, it is mandatory to conduct the studies with substitute materials, so-called lunar regolith simulants. The properties of lunar regolith in terms of chemical and mineral composition, as well as particle size distribution and the geotechnical properties of different landing sites, are well known from the Apollo and, to a lesser extent, Luna missions [30,33,34,35,36] and can be recreated very precisely by terrestrial materials.

### 1.3. Regolith Processing Technologies

Knowing the lunar surface properties is not only important for rover maneuvering. Various ISRU methods and processes for the processing of lunar regolith as a building and source material have already been researched. Due to its fineness (soil < 1 cm, dust < 20 µm), lunar regolith is an excellent construction material, because the particles do not need to be ground but only mechanically sieved [28]. Current efforts not only deal with the production of technical equipment, tools, and spare parts but also with high-mass components such as roads, buildings, launch, landing pads [37], and habitats or shelters from lunar regolith [8,9]. Since the dimensions of lunar outposts and the required structures remain unknown, the building technologies should be flexible [4], providing safety by being able to quickly repair failed systems [38,39].

Additive manufacturing technologies are particularly suitable for the highly efficient and cost-intensive aerospace sector because they allow the realization of complex structured components with minimal waste production and pre/post-processing effort [40,41]. To avoid the use of binders, the interest in processes that use the raw lunar regolith increased. Well-known methods are sintering-based additive manufacturing technology [42,43] like high-powered lasers, microwave [42], or solar sintering [44]. Other additive manufacturing techniques, such as selective laser melting (SLM) [8,45], mobile selective laser melting (M-SLM) or powder-feed fused deposition modelling (PF-FDM) [46] have been further examined. However, processes incorporating binders and additives are also relevant. Utilizing feedstock from previous exploration missions helps to close the material cycle. Lunar concrete was first proposed in 1985 [47], and since then it has been further developed by many research groups with regolith samples from Apollo missions as well as analog lunar materials [48,49,50,51,52,53]. Newer approaches include lithography-based ceramic manufacturing (LCM) [54] and the use of thermoplastics as a matrix material [55]. On the other hand, using regolith as a casting material has also been investigated [56].

For all of these technologies, lunar regolith must be excavated, transported, and processed. Digging [57] and drilling [58] are not only important for the sampling process but mandatory for all the above-mentioned technologies. These must be designed and optimized for the materials involved. Hence, terrestrial soil mechanics must be well understood.

### 1.4. Lunar Regolith Simulants and Their Requirements

The returned regolith samples of the Apollo missions were used to extensively characterize their chemical, physical, and, as far as possible with the amount of material available, their geotechnical properties. In order to give manufacturers and users guidelines, a list of 32 important characteristics that are considered crucial for the research and development of lunar applications was developed by a committee of experts during the 2005 Lunar Regolith Simulant Workshop held by MFSC [59]. To date, these specifications are the most current and accurate in existence. The properties were then ranked due to their importance, from which the following eight most important factors emerged (in decreasing order of weighted importance): particle size, particle size distribution, particle density, glass composition, bulk density, mineralogical composition, particle shape, and bulk chemistry. Hence, after a compositionally suitable terrestrial material was found, it is important to process and characterize it by using appropriate technologies in order to ensure that the simulants’ geotechnical and (geo)mechanical properties resemble the original as close as possible.

Since the Apollo missions, many different simulants have been developed worldwide, but most of them are only provided on a small scale and are designed for specific applications. Some of them were made available in large quantities for worldwide research, although commercially available simulants mainly simulate mare soil, as most Apollo missions took place there [4]. Well-known representatives are, e.g., JSC-1 with its variations JSC-1A, JSC-1AF, or JSC-1AC, which simulate low-Ti sites of the basaltic bedrock and are used for geotechnical applications [60]. MLS-1 and MLS-1P (high Ti) also simulate mare regions and are mainly used for general purposes [60]. EAC-1 was developed and manufactured in Europe and is used in geotechnical areas [61]. Other mare simulants include the Japanese FJS-1 (low and high Ti) [60] or the Chinese CAS-1 and CLRS-1 and -2 (general; low and high Ti) [62]. With MLS-2, OB-1 [60] or NAO-1 [62], there are also analog materials for highland characteristics for various applications. Newer analogous materials include UoM-B and UoM-W, which do not mimic any particular region. Nevertheless, they are suitable as low-fidelity simulants for large experimental setups [63]. Other large-volume simulants are the basanitic EAC-1 and its dust-free version EAC-1A [64]. LHS-1 and LMS-1 are meant to mimic both mare and highland regions [65]. Further, other regolith simulants exist worldwide.

### 1.5. Adaptable Lunar Regolith Simulants TUBS-M, TUBS-T, and TUBS-I

So far, no single lunar simulant can cover the multitude of use cases that lunar regolith involves [62]. To overcome this major gap, the Institute for Particle Technology and the Institute of Space Systems (IRAS) at TU Braunschweig have collaborated to develop, produce, and investigate various lunar regolith analogous materials. A modular system was developed to meet general, as well as more specific demands, enabling the simple and reproducible production of different lunar simulants in small amounts. This allows, for example, to recreate regolith samples of specific landing sites or sampling locations. For this purpose, three types of simulants have been identified [1,8]:

1. Base simulants: They consist entirely of lithic particles and are suitable for general applications, simple processing experiments, and geotechnical investigations.
2. Chemically adapted simulants (CAS): Compared to base simulants, CAS additionally contain different proportions of minerals found in lunar rocks like ilmenite or olivine. This type is particularly suitable for the extraction of chemical components within the scope of in situ resource utilization (ISRU) research or for the production of thermally altered particles such as agglutinates and glasses.
3. Physically adapted simulants (PAS): They contain agglutinates and glass particles, which were produced from CAS by thermal adaptation processes. This type can be used for advanced and highly specific applications.

Although the lunar surface is mineralogically and chemically very diverse, basalt and anorthite provide the foundation for the two base artificial lunar soils: the mare simulant TUBS-M and the terrae simulant TUBS-T. The prefix “TUBS” stands for Technical University of Braunschweig. Since they occur as a mixture in most regions of the Moon’s surface, the “intermediate” simulant TUBS-I was additionally introduced and investigated. It consists of a 50:50 mixture of TUBS-M and TUBS-T. Within the scope of this study, the geo-, bulk solids-, and particle-technical, as well as the chemical properties, of these three base simulant soils, are discussed. CAS and PAS regolith simulants will be presented in future publications.

### 1.6. Mission Risks and Relevance of Data Acquisition

Space exploration missions are dangerous and demanding endeavors that face unique technological challenges and hazards that demand thorough risk assessment prior to any mission. Whether it is about the return to or colonization of the Moon, Mars, or other celestial bodies, they all include both human and robotic exploration activities. Human health and performance risks are crucial for long-duration space travel. The NASA Human Research Program (HRP) has identified over 30 human health risks associated with missions to Mars [66], including factors such as space radiation [37], altered gravity fields, isolation and confinement, the closed environment, and distance to Earth. On a more technical side, the most risky phase of manned missions is entry, descent, and landing (EDL) [67,68,69]. Surface knowledge and terrain-based planning are considered critical key factors to ensure mission success [70,71]. By knowing the soil’s geotechnical properties, terrain hazards that compromise the operation of rovers or ISRU equipment can be avoided [72,73,74,75]. All of these risks, findings, and precautionary measures are not only relevant for explorations to the Moon, but for Mars or every other celestial body, only the strategies might differ [75].

Every endeavor that involves celestial-body exploration inevitably deals with bulk material. While there has been research on using simulant materials to examine various applications and prepare lunar missions, the majority of them was performed with a minimum amount of output data regarding the used lunar simulants. Often crucial information cannot be found in the literature. However, the reliability of the output data based on it depends on the accuracy and knowledge of the material properties and their often very diverse interactions with the technologies examined.

The aim of this study is to present a novel contribution to this issue by further characterizing the so far unique flexible adaptable lunar regolith simulants TUBS-M, TUBS-T, and TUBS-I [1,2]. Comprehensive investigations regarding their geotechnical properties and soil-based mechanics are provided and their influence on various technologies and mission risks are discussed and evaluated.

## 2. Materials and Methods

### 2.1. Processing of the Raw Material

Experiments with the three lunar regolith simulants were conducted: TUBS-M, TUBS-T, and TUBS-I. The raw material for the two lunar simulants, TUBS-M and TUBS-T, consists of pea gravel with grain sizes of up to a few millimetres. Information on the origin of the raw materials can be found in previous papers [2]. Lunar regolith has a broad particle range from several nanometers up to over 1 mm [76].

**Figure 1:**
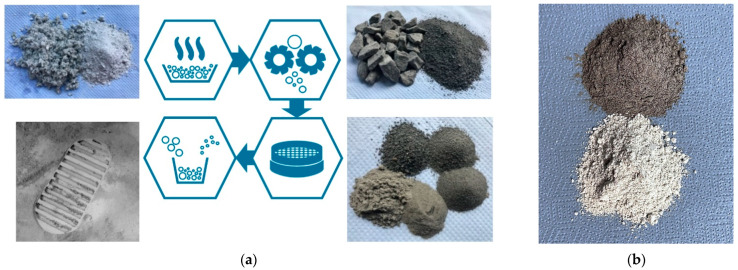
Process chain of regolith simulant production (**a**) and photographs (**b**) of TUBS-M (top) and TUBS-T (bottom) after the final mixing step.

The materials were processed through a process chain as illustrated in Figure 1a. Firstly, the crude material was dried in an oven (UL 80; Memmert GmbH & Co. KG, Schwabach, Germany) at 100 °C for several days. Coarser particles (>2 mm) were then ground in a dry operated universal turbo mill (UTO2, Bauermeister Zerkleinerungstechnik GmbH, Norderstedt, Germany) that is equipped with a sieve insert with 2 mm hole diameter. The material was split into fractions using a vibro-energy separator (S24S; SWECO, Florence, KY, USA) for grains down to 250 μm, while “fraction” describes the particles remained between two sieve trays.

**Figure 2:**
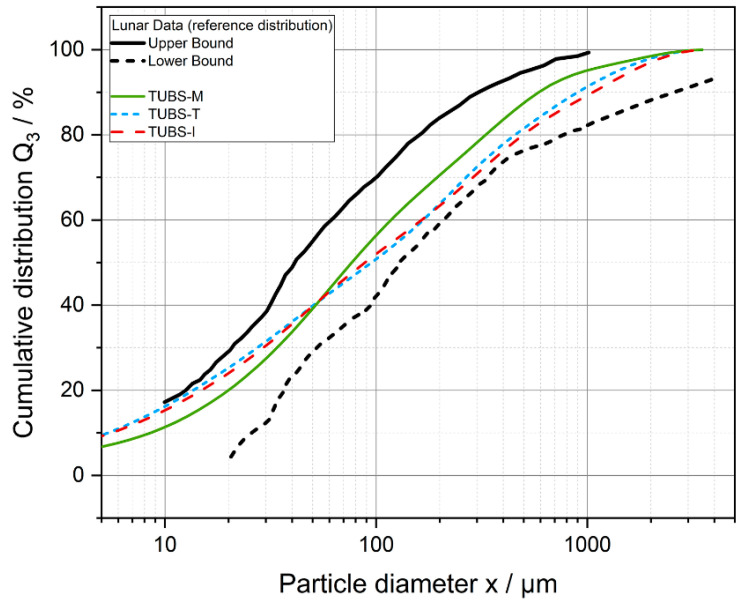
Particle size distribution (measured by Mastersizer 3000, Malvern Panalytical GmbH, Kassel, Germany) of the examined batch samples of TUBS-M, TUBS-T, and TUBS-I in the distribution band for Apollo 11, 12, 14, and 15, modified from [76]. Particle diameter equals particle size.

Sieving aids were necessary to precisely separate finer and coarser particles into individual fractions. Hence, grains below 250 μm were processed using an air jet sieve (e200 LS; Hosokawa Alpine AG, Augsburg, Germany) for small samples and a tumbler screening machine (TSM 1200, Allgaier SE, Munich, Germany) for larger quantities. Afterwards, the fractions were mixed together at appropriate proportions to obtain a sample corresponding to the particle size distribution defined in Figure 2. For this purpose, a 3D shaking mixer (TURBULA, Willy A. Bachofen AG, Muttenz, Switzerland) was used for small quantities and a conventional concrete mixer for larger sample masses. Figure 1b shows a TUBS-M and TUBS-T sample after the final mixing step. The proportions were calculated using a tool developed at the Institute for Particle Technology. It considers the distribution of the individual particle size fractions in a broad distribution. By summing up the fraction proportions and varying them, mixing ratios are obtained.

The results of this adjustable process chain are base simulants that entirely consist of lithic particles to suit general applications, geotechnical investigations, and simple processing experiments such as sintering. Figure 2 describes the cumulative fraction Q_3_ of the particle sizes for TUBS-M, TUBS-T, and TUBS-I samples determined by laser diffraction (Mastersizer 3000, Malvern Panalytical GmbH, Kassel, Germany) compared to the distribution band that is derived from samples of various Apollo missions. Since the majority of lunar regolith samples lies within a narrow grain size range, as reference the base simulants grain size distribution was selected [76]. However, the particle size distribution of different batches can always differ slightly. The results also depend on the particle size measurement system used.

### 2.2. Particle Size Distribution Measurement

The particle size distribution (PSD) is an important factor, since it directly influences a lot of geotechnical properties such as the shear behavior (e.g., cohesion or flow), permeability, compaction, and porosity. Conventional methods to determine the PSD are, for instance, sieve analysis (ASTM D422, 2005) [77], laser diffraction [78,79], X-ray microtomography [80], and dynamic/static image analysis [81].

Since the PSD of the lunar simulants ranges from a few micrometers to over 1 mm, sample preparation prior to particle size measurement is mandatory. Widely distributed materials always show segregation behavior, leading to irregularities in the particle size composition depending on the sampling location and type. This is why the materials were prepared with a sample separator to gain representative samples of the initial material and to ensure the reproducibility of the results. A sample divider PT 100 (RETSCH GmbH, Haan, Germany) and a sample splitter RT 25 (RETSCH GmbH, Haan, Germany) were used for small quantities and large batches of up to 25 kg, respectively. The PSD was then measured via laser diffraction analysis (Mastersizer 3000, Malvern Panalytical GmbH, Kassel, Germany), using the dry dispersion unit Aero S at an air pressure of 2 bars. The obtained data was evaluated applying Fraunhofer theory. In order to compare the results of particle-size analyses with different systems, additional measurements were carried out using a QICPIC (QICPIC/L + GRADIS + VIBRI/L, Sympatec GmbH, Clausthal-Zellerfeld, Germany) and sieve analysis according to DIN 66165-1. If not further mentioned, the results shown are determined by laser diffraction with the Mastersizer 3000 (Malvern Panalytical GmbH, Kassel, Germany).

### 2.3. Measurement of Particle Morphology and Surface Structure

The aspect ratio was analyzed by a dynamic image analysis system (QICPIC, Sympatec GmbH, Clausthal-Zellerfeld, Germany) with the wet LIXELL unit in water. Lastly, the fine-grained surface textures of the lunar regolith simulants were studied with a scanning electron microscope (SEM; Helios NanoLab G3 UC, FEI Deutschland GmbH, Dreieich, Germany) in secondary electron mode (acceleration voltage set of 5 kV). The samples were placed on carbon tape discs and coated with 4 nm Pt-coating.

### 2.4. Bulk and Flow Properties

In order to obtain information about the stress–strain behavior of the consolidated simulant materials, ring shear tests were performed (RST-01; Dr. Dietmar Schulze GmbH, Wolfenbüttel, Germany). The measuring procedure is well known and defined in the literature [82,83,84,85,86,87]. The shear cell used has a specimen volume of 215.36 cm^3^, and the lid has lamellar carriers. Since testing stresses for direct shear tests in literature show a wide range from 1 to 300 kPa (in triaxial tests, up to 625 kPa) [77] and considering that the gravity on the Moon is about one-sixth of that on Earth (*g* = 1.62 m/s^2^), relatively low shear stresses of 3, 6, 9, 15, and 25 kPa were used to perform the tests. These values refer to low and near-surface depths on the lunar surface.

The bulk density *ρ_B_* was experimentally determined according to ISO 697. The tapped density *ρ_T_* and, thus, the compaction behavior, was assessed with a tapping device according to DIN EN ISO 3953. Consequently, a volumetric analyzer (SVM 121, ERWEKA GmbH, Langen, Germany) with 2880 movements was used. The powder volume was recorded as a function of the number of taps *N* until *N* = 500 was reached, above which no further densification could be observed in any sample. The tapped density was then obtained from the volume measured after 500 taps.

## 3. Influence of Measurement System on Particle Size Distribution

### 3.1. Methodological Background of Relevant Measurement Procedures

The particle size distribution (PSD) describes important physical properties that strongly influence the behavior of bulk solids. Thus, it is of utmost importance that the particle properties are known with sufficient accuracy for many scientific and technical questions. The most common measurement methods are sieve analysis, light scattering (LS, also laser diffraction), and dynamic image analysis (DIA). Each of these methods covers a characteristic particle size range in which measurements are possible, although these ranges partly overlap. Even though particles are measured in a range that is characteristic for all of these systems, the competing measurement techniques often provide significantly different results, depending on the physical measurand under consideration. Therefore, it is important to understand this principle and to choose and document appropriate measurement parameters. A comparison of different studies can solely be ensured and deviation interpreted correctly, if the aforementioned applies. In the following, the three most frequent and for this study relevant measuring methods are described in detail.

The sieve analysis is the most commonly used particle size measurement and represents a fractionating method. Hereby, the particle system is classified with regard to the cross-section area of particles, and in a second step, the quantities of the individual size fractions are evaluated. A conventional sieve analysis involves separating the sample over several test sieves with increasing mesh sizes and then weighing the fractions. The fractions are converted into a mass-weighted size distribution [88]. Ideally, the particles pass the smallest possible sieve mesh with their smallest projection areas. Therefore, the sieve analysis is mainly determined by the particle cross-section and thus influenced by the particle shape. The measurement is time-consuming and can barely be automated, and even small errors and wear of the sieve meshes falsify the result.

During dynamic image analysis, particles are guided passing a camera system and analyzed in real-time; thus, modern equipment can record several hundred images per second, with a measurement result consisting of the evaluation of several hundred thousand to several million particles. Unlike sieve analysis, DIA measures particles in a completely random orientation. Their projection area is considered, leading to differences in the measurement results, being characteristic for each specific particle shape. By means of adjustment algorithms, the results can be correlated very closely with analytical sieving, thus ensuring good comparability between measurements with different systems. In addition to size, the resulting particle images can be used to determine shape parameters such as aspect ratio, convexity, or symmetry. DIA systems are designed for analyses in the micrometer range, wherein they can detect even small differences in particle size and reliably detect multi-modal distributions. However, the pixel resolution is 1–2 µm, which is why fine particles below 10 µm often cannot be reliably analyzed [89].

Laser-scattered analysis is a spectroscopic measuring method in which the PSD is derived from the scattered laser signal. The particle sizes are measured only indirectly by detecting the intensity patterns of scattered light at different angles. The calculation of the PSD is conducted either by Mie theory or by Fraunhofer approximation [88]. The Mie theory is based on the measurement of the scattering of electromagnetic waves on spherical particles. Therefore, no shape evaluation is possible. It resolves most reliably in a fine size range below several µm. The refractive and absorption index of the sample material must be known for a meaningful measurement. The Fraunhofer approach is a simplified approximation that does not require knowledge of the optical properties of a sample and provides more reliable results above a particle size of approximately 5 µm than the Mie theory [90]. Furthermore, this approach does not assume a spherical particles, so shape parameters can be taken into account. The advantages of LS systems are that they are fast, well-established, automatable, and versatile and can measure particles in a broad size range. However, the multi-modality of polydisperse samples can only be resolved to a limited extent due to the overlapping scattered-light signals.

### 3.2. Particle Size Measurement of TUBS-M

**Figure 3:**
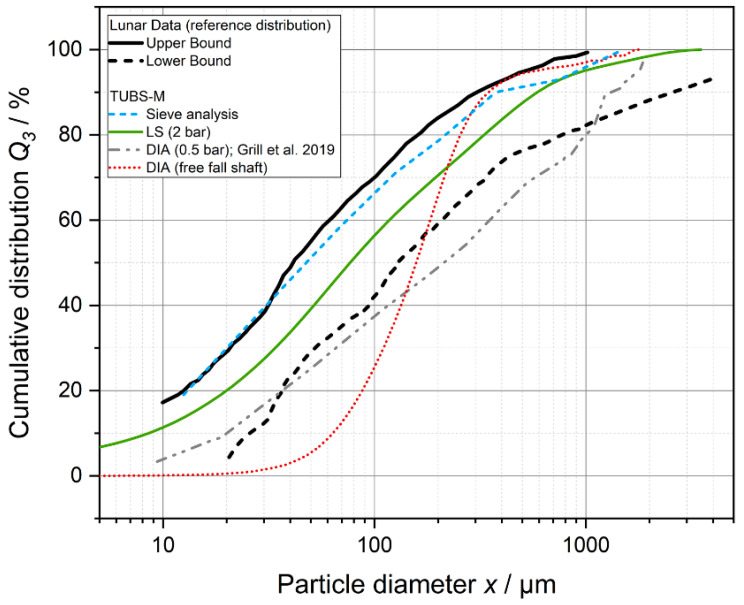
Cumulative particle size distributions of TUBS-M determined with different methods and from different authors (also Grill et al. [81]) compared to literature reference data of real lunar regolith [76]. Particle diameter equals particle size.

The same lunar regolith simulant systems are repeatedly measured, compared with each other, and used for various investigations in the literature. Subsequently, method-specific measurement deviations are explained using the regolith simulant TUBS-M. For this purpose, TUBS-M was analyzed with the previously described methods and plotted together with the distribution band from lunar regolith data in Figure 3. Due to the extraterrestrial application, all samples were measured in dry condition without a liquid dispersing medium. Three samples each were prepared in small scale (50 g) and were mixed together in the respective ratios from the previously sieved fractions. Hence, samples were not taken from a single batch. Each sample was determined threefold. In addition, the cumulative distribution measured for TUBS-M by Grill et al. [81] was discussed, which was determined using DIA (CAMSIZER X2, Microtrac Retsch GmbH, Haan, Germany).

**Figure 4:**
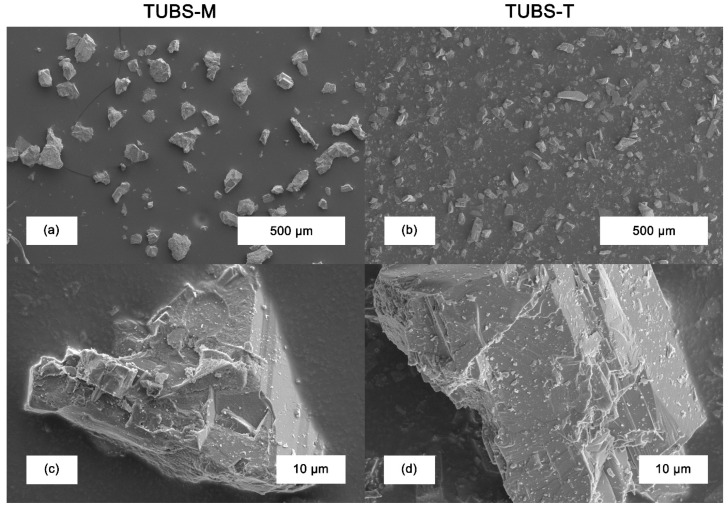
TUBS-M (**a**) and TUBS-T (**b**) particles of the size fractions 45–90 µm and 25–45 µm, respectively; surface structure of a single particle of the size fraction <25 µm for TUBS-M (**c**) and 25–45 µm for TUBS-T (**d**).

The reference value of TUBS-M, as given by the authors in [2], was determined by laser diffraction. The samples were dispersed in a Venturi nozzle at 2 bars of pressure in a compressed air stream. The cumulative distribution according to Grill et al. [81] shows a shift towards larger particle sizes, whereas the curvature is very similar to the reference. The shift is mainly caused by the lower pressure of the dispersion air stream of 0.5 bar. Smaller particles still tend to stick to the surface of coarser particles due to strong Van der Waals forces. Therefore, the fraction of fine particles <10 µm can not be counted by the system and is completely missing in the DIA measurement, leading to the impression of a shift towards larger particle sizes. Similar effects can be seen in the DIA analysis performed in the context of this study using a free-fall shaft without applying dispersing air pressure. The distribution deviates strongly from the two discussed curves and hardly shows any fine particles <100 µm. The DIA method using a free-fall shaft is particularly suitable for free-flowing powders and illustrates the cohesiveness of TUBS-M: Due to particle–particle interactions, the broad PSD and different shape factors, the particles adhere strongly to each other and form large agglomerates. They cannot be separated without appropriate dispersion units, which greatly distorts the measurement result towards large particle sizes. The sieve analysis shows the same trend as the distribution band but approximates the upper limit. This shift towards a higher proportion of small particles could be explained by the fact that elongated particles pass through the sieve meshes with their short sides and appear as fine particles. The morphological diversity is demonstrated in Figure 4.

Using these exemplary measurements, considerable differences in the resulting data can be identified. Ultimately, the choice of the measurement system depends on the properties to be investigated. The determination of the PSD of cohesive lunar regolith and/or simulant samples usually involves the separation and measurement of the primary particle sizes. In this study, it is shown that the measurement method of DIA in the free-fall shaft is not suitable. For dry measurements, a sufficiently strong air or gas flow must be introduced into the system. Wet measurements are also conceivable, but it must also be ensured that the samples are sufficiently dispersed in order to exclude the distortion of the measurement results due to sedimentation effects. In addition, if wide PSDs are present, attention must be paid to obtain representative samples by prior sample splitting. Basic regolith simulants, such as TUBS-M, which are based on the distribution band according to Carrier et al. [76] (determined by sieve analysis), consist of approx. 80% particles <300 µm. Therefore, even small deviations of coarser particles have a significant influence on the PSD, which, however, have no or only a marginal influence on the resulting bulk properties investigated in this study. In this context, care must be taken with LS measurements, which do not sufficiently resolve coarse particles >500 µm if the measurement parameters are not set appropriately.

## 4. Properties of Lunar Regolith Simulants

### 4.1. Chemistry and Morphology of Lunar Regolith and Simulants

TUBS-M basalt is dark gray and has a homogeneous, fine-grained crystal structure. It corresponds to typical basalt. The size and shape of the simulant particles produced from that material is influenced by the characteristics and the fracture properties of the crystals. Due to the heterogeneous mineral composition of the basalt, sharp-edged and irregular particles, needle-shaped particles, and almost round particles occur. The chemical composition is dominated by the oxides SiO_2_, Al_2_O_3_, FeO, MgO, and CaO, from which the main mineral phases plagioclase, pyroxenes, and olivine, are formed. In the case of minor components, such as TiO_2_, MnO, and Cr_2_O_3_, the measured values are in the low range (<1%), which means that the TUBS-M basalt contains no rarer mineral phases in large quantities.

Anorthite (TUBS-T) has a light color and a dense, fine-crystalline structure with larger plagioclase crystals embedded. Some areas are colored gray, indicating the presence of additional mineral phases next to the plagioclase. These are formed by metal-containing mineral phases, which are called mafites. Compared to basalt, anorthite is an almost monomineral rock, as it consists of over 90% of feldspars from the plagioclase group. Accordingly, the chemical composition of the TUBS-T anorthite is characterized by the oxides SiO_2_, Al_2_O_3_, CaO, and Na_2_O. The Na content results in the formation of the plagioclastic type bytownit ((Ca, Na) [(Si, Al)_4_O_8_]), which dominates the rock, with 97 vol.%. Other oxides occur to a small extent, for example TiO_2_ (0.12 wt.%), FeO (1.05 wt.%), MgO (0.57 wt.%), and K_2_O (0.22 wt.%). The high proportion of feldspars leads to the formation of tabular particles with sharp edges during the simulant production process.

Note that TUBS-I is a 50:50 mixture of TUBS-M and TUBS-T, and the mineralogical and chemical composition is correspondingly average. Further details on the mineralogical and chemical composition of the simulants can be found in the previously published works of the authors [2].

The different chemical compositions lead to the formation of individual mineral phases, whose crystals have different fracture behavior, which results in the development of a variety of particle morphologies. In Figure 4, SEM images of TUBS-M and TUBS-T particles of different size fractions are shown. Figure 4a,b illustrate the very diverse primary particle morphologies that occur, ranging from elongated to almost equal-sided, compact particles. Figure 4c,d further emphasize these observations, because the multitude of fracture surfaces leads to the development of different surface structures. Feldspars typically break along their preferred directions (“plate-like” fractures), which is noticeable from the plagioclase bytownite present in TUBS-T in Figure 4d. In contrast, many other minerals mainly break irregularly, as shown in Figure 4c for a TUBS-M particle.

The breakage behavior and therefore the aspect ratio significantly influence all other bulk material properties. In addition, particle shape plays an important role in damage mechanisms. Sharp-edged particles are more likely to pierce through material (e.g., rover wheels) [72] and cause stronger abrasion on all regolith processing equipment. Diverse morphologies lead to fluctuating properties, which creates challenges in the conveyance of regolith and thus affects the design of equipment.

Furthermore, in all samples, it can be observed that fine particles loosely adhere to the surface of coarser particles. This results from the sieving process during simulant production. Very fine particles strongly interact with the surface of larger particles due to attractive Van der Waals forces and cannot be removed by the dry process. These nano-grains are part of the lunar dust, which is described as clinging, penetrating, abrasive, and resource-rich [91]. Its stickiness causes huge problems, and system-wide dust protection is a key design driver for the development of dust-proof mechanisms, bearings, materials, and coatings and is considered critical for mission success [92].

### 4.2. Particle Size Fractions in the Broadly Distributed Lunar Regolith

**Figure 5:**
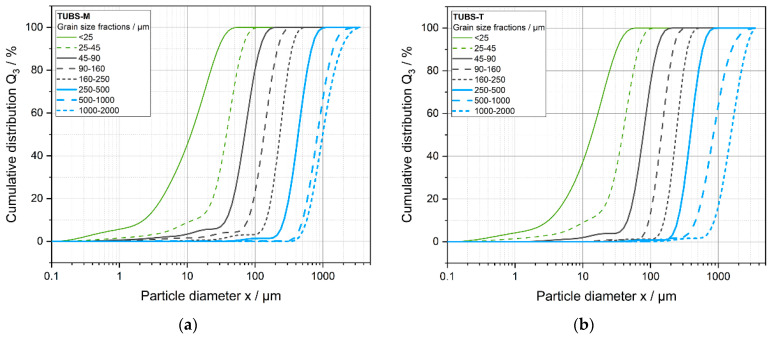
Cumulative particle size distributions of the grain size fractions of TUBS-M (**a**) and TUBS-T (**b**) measured by LS. Particle diameter equals particle size.

In order to understand how the broadly distributed lunar regolith behaves, it is advisable to investigate different size ranges separately in order to better explain observations on the original mixture (containing the full PSD). In this work, the lunar regolith simulants TUBS-M and TUBS-T were divided into eight size fractions, whose particle size fractions (PSDs; measured by LS) are shown in Figure 5. Since TUBS-I is a mixture of TUBS-M and TUBS-T, it was not considered in this part of the investigation and is discussed in Section 4.3.

The distributions of the individual fractions are very narrow, indicating a high selectivity, whereby oversized and undersized grains occur in all fractions in small proportions. The width of a PSD can also be described by the polydispersity index (*PDI*). It is defined by *PDI* = (*x*_90_* − x*_10_)/*x*_50_ and reflects the dispersity of colloidal systems. A *PDI* of 0–0.3 indicates an approximately monodisperse system, whereas a *PDI* > 0.5 indicates a polydisperse system. The latter applies to almost all fractions of TUBS-M and TUBS-T. The exact numbers are listed in Table 1 accompanied with the lower, mean, and upper values of the size distributions.

It can be observed that the *PDI* tends to decrease with increasing particle size, as the separation efficiency of the sieves becomes sharper with increasing grain size. This is the consequence of stronger adhesion of fine particles due to higher specific surfaces and increased tendency to agglomeration.

Further, a small amount of fines is present in most of the coarser fractions. The greater the percentage of undersized grain remaining, the further the *x*_10_ value (see Table 1) deviates from the lower screen cut. The presence of fine particles could already be observed and discussed in Figure 4 and is confirmed by these measurement results. During the particle size measurement by laser diffraction, the particles are dispersed in an air stream with compressed air at 2 bars of pressure. The fine particles adhering loosely to the surface of larger particles are detached and can now be detected by the measuring system. Building on this, Figure 5 shows the major lunar dust problem discussed in the previous chapter. In terms of particle count, dust-sized particles can be found in significant amounts in all fractions.

**Figure 6:**
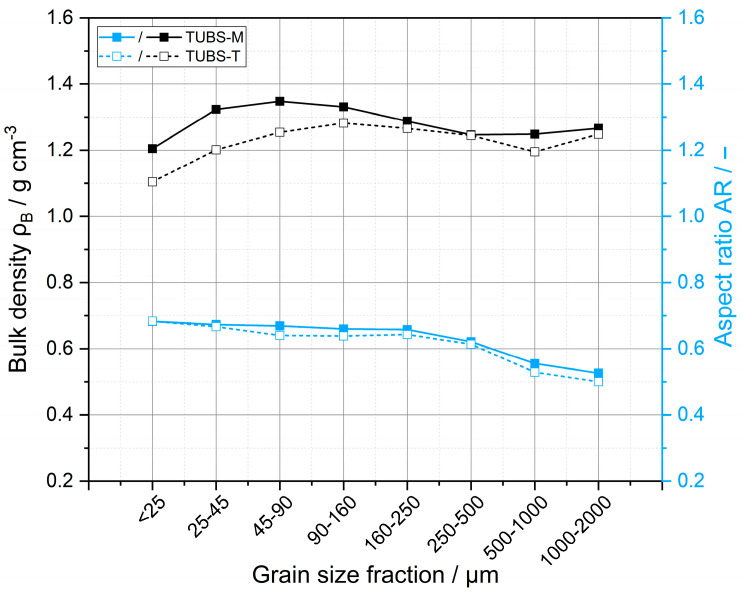
Average aspect ratio and bulk density of the grain size fractions of both simulant materials.

In addition to a visual analysis, particle shapes can be described by the aspect ratio. It is calculated using the Ferret diameter as the quotient between the shortest and longest particle diameter *D_Feret_* = *x_(F,min)_*/*x_(F,max)_*. The aspect ratio ranges from 0 to 1, wherein 0 describes a perfectly elongated and 1 a perfectly spherical particle. Smaller particles tend to form rounded structures due to their geometry. This is demonstrated in Figure 6 by a decreasing aspect ratio with increasing particle size. The particles of TUBS-T also show a slightly more pronounced angularity in all fractions. Constant meteoroid bombardment and the absence of hydrospherical and atmospheric erosion result in the formation of distinct angular shapes, which are highly abrasive and cause significant engineering challenges [93].

There is little data available on the aspect ratio of real lunar regolith. Görz et al. [94,95] examined 2066 particles in the size range of 1.25–30 µm and reported that most values fall in the range of 0.4 to 0.7 (slightly-to-moderately elongated), which results in an average aspect ratio of 0.55. The particle shapes are so diverse that the average value can only be regarded as a rough guideline. The irregular and angular particle shapes of both TUBS-M and TUBS-T allow experiments under realistic conditions.

### 4.3. Bulk Density and Consolidation

The flow and compaction characteristics of bulk solids are key parameters that are essential for the design of mobility systems or excavation equipment. They are influenced by many factors, such as particle shape, size distribution (PSD), and temperature or chemical composition, as well as interparticulate interactions, such as cohesion/adhesion or frictional forces [96]. Thus, a bulk material behaves like a liquid in a fluidized state (e.g., on the powdery lunar surface in low gravity) and like a solid in a compacted state (e.g., several cm below the surface). In reality, all states between these boundary conditions occur.

The bulk densities of the individual size fractions were already plotted in Figure 6. It is shown that even though the bulk densities of TUBS-M (1.20–1.35 g cm^−3^) and TUBS-T (1.10–1.28 g cm^−3^) are very similar, the former tends to achieve higher bulk densities due to more spherical particle shapes. On the other hand, the PSDs within all fractions of both materials are very narrow (see Figure 5), which gives the bulk material homogeneous properties overall. However, a tendency towards lower densities can be seen from 90 μm onwards. The fine-particle fractions contain a higher number of agglomerates, which contribute to forming large voids in the packed-bed due to their strong particle–particle interactions. The coarser fractions also show a tendency towards lower bulk densities, caused by the fact that coarser particles can only rearrange themselves to a limited extent in the measuring cylinder. Consequently, larger pores, and thus, lower densities remain in the bulk bed. The exact values for the measured bulk density for all size fractions are listed in Table A1 together with the tapped density.

**Figure 7:**
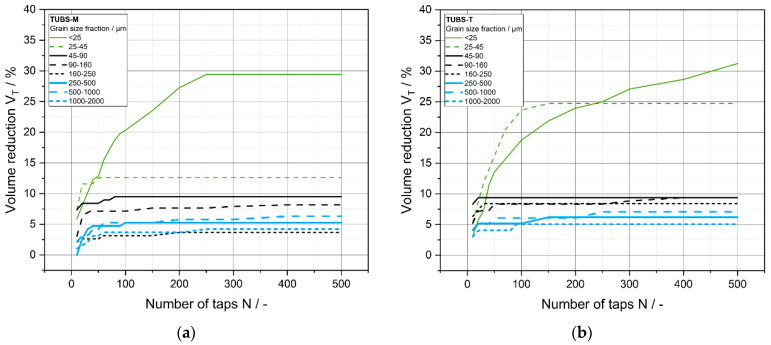
Volume reduction of the grain size fractions of TUBS-M (**a**) and TUBS-T (**b**) as a function of the number of taps.

The solidification behavior of bulk materials can further be described by using compaction tests. The bulk density represents the initial state of the tapped density at time *t* = 0, before any densification occurred. Figure 7 describes the solidification behavior of the different size fractions of TUBS-M and TUBS-T by tapping. Thereby, the volume reduction of the starting volume in a measuring cylinder is plotted as a function of the number of taps. The slope of the curves demonstrates that the pore volume decreases rapidly at the beginning of the compacting process for both materials. For TUBS-M, this area extends to about 50 taps and for TUBS-T only to around 25 taps, which implies that the simulated materials densify strongly under little external influence. This is especially crucial for the safety of planetary rovers, as terrain properties can quickly change. For instance, the Mars Exploration Rovers Spirit and Opportunity got stuck in soft soil [72]. However, any regolith-processing equipment could become clogged or damaged as a result. For the setup of infrastructure and landing pads, the fast densification can be used as an advantage. Considering the low gravity on the surface of the Moon (1.62 m/s^2^), lunar dust formation and migration plays an even bigger role, as there are large numbers of fine particles and they settle only slowly. Furthermore, lunar dust is known to become highly electrostatically charged, sticking to surfaces and thus, damaging equipment [97] and affecting lunar rover movement [28,98,99,100].

**Figure 8:**
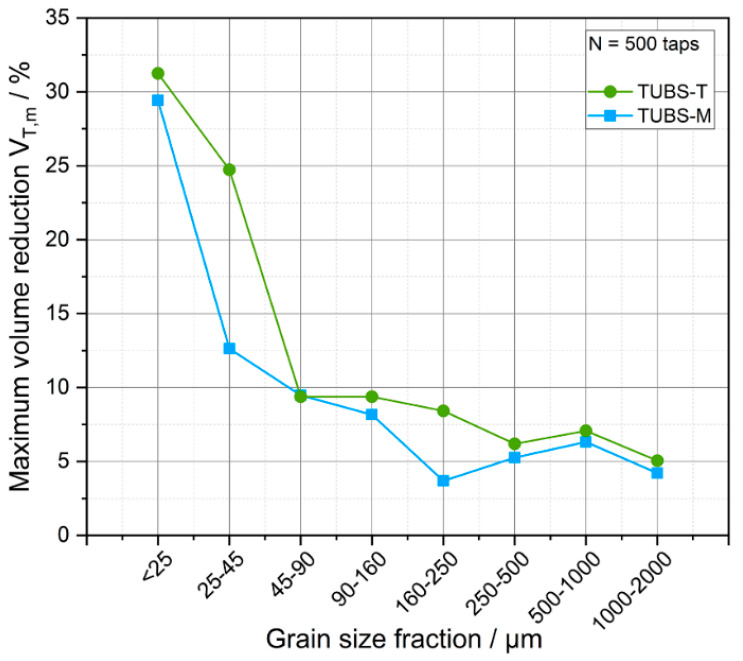
Maximum volume reduction at *N* = 500 taps as a function of the particle size fractions.

Up to the 90–160 µm size fraction, the densification values follow a pattern: the finer they get, the stronger the volume reduction, whereas the coarser fractions do not follow a specific pattern and instead fluctuate. The reason for this is that, although the samples have the same initial volume, the number of particles in the measuring cylinder is much smaller for the coarser fractions. Therefore, they have less of a possibility of rearranging themselves. In addition, larger cavities remain between the particles in coarser fillings, which means that these fractions are less dense, as they tend to form a loose, free-flowing packing. Lower interactions coupled with a higher weight force result in a loose packing skellet whose density hardly changes. For particle sizes <90 µm, the influence of particle–particle interactions compared to the weight force becomes clearly visible through more pronounced volume reductions. This is illustrated in Figure 8, shown by the tapped density at full compaction (*N* = 500 taps). Therefore, the tendency for stronger compression at lower particle sizes is clearly illustrated. The overall more fluctuating properties of TUBS-T are related to its mineralogy and fracture behavior (see Section 4.1). Its lower aspect ratios (see Figure 6) and different surface properties (see Figure 4) contribute significantly to the total porosity of the bed. Particles rearrange more often and maximum densification is achieved later, a factor that becomes more pronounced the smaller the grain sizes are.

Another explanatory approach is the *PDI* (see Table 1): the higher the *PDI*, i.e., the more polydisperse and inhomogeneous the system, the denser the packing can become. All these observations show that small particles are most influential on the engineering response of the terrestrial and therefore lunar soils [101].

### 4.4. Shear Tests and Flowability

In addition to tap densification, the solidification behavior of bulk materials can be described by their flowability. To characterize the flow behavior of bulk solids with a ring shear tester, the ratio *ff_c_* of consolidation stress *σ*_1_ to unconfined yield strength *σ_c_* according to Jenike [82] is frequently used, which is defined as *ff_c_ = σ*_1_*/σ_c_* and called the flow function. The higher the *ff_c_*, the easier a bulk material flows.

**Figure 9:**
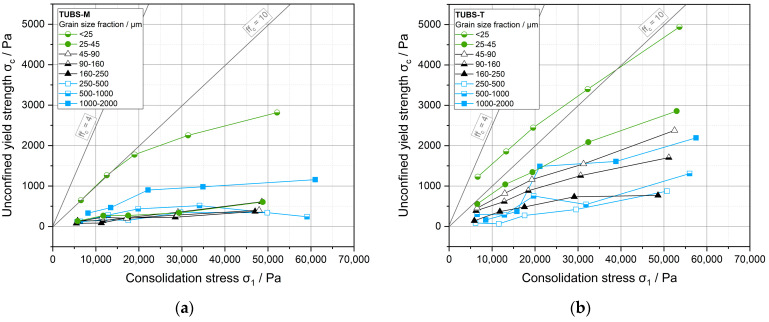
Flow functions of the grain size fractions of TUBS-M (**a**) and TUBS-T (**b**) at shear stresses of 3, 6, 9, 15, and 25 kPa.

In Figure 9, the flow functions of all investigated particle size fractions of both TUBS-M and TUBS-T are depicted. The individual data points on the flow functions are the maximum normal stresses acting on a free-standing bulk solid before it breaks due to shear. This unconfined yield strength *σ_c_* is achieved from a so-called flow locus presenting the dependency of shear stress for beginning flow as a function of normal stress [102,103]. This point is frequently described as uniaxial compressive strength obtained from uniaxial compaction testers [104]. When reviewing the literature, it is important to note that the results will not be directly comparable because of different stress state conditions in these two measurement systems [105]. The location of a flow locus depends on the previous consolidation stress history: The more pronounced it is, i.e., the higher the consolidation stress *σ*_1_, the higher the bulk density and the higher the strength of the bulk solid.

For both regolith simulants, the particle size fractions >25–45 µm show easy to free-flowing behavior, whereas TUBS-T in comparison displays slightly poorer flowability. According to Schulze [106], particles of the same size range flow worse

- the lower their aspect ratio is,
- the smaller the porosity in the bulk material is and
- the greater the friction coefficient between the particles.

As already shown in Figure 6, the TUBS-T grains have a slightly lower aspect ratio, which can lead to poorer flow properties due to particle distortion. The particle size distributions <25 µm (see Figure 5) are almost the same for both materials so that an influence of this can be excluded. Furthermore, the maximum volume reduction in percent gives conclusions about the porosity of a bulk material. In Figure 8, all size fractions of TUBS-T show a higher maximum volume reduction after 500 taps, which implies a lower porosity, leading to poorer flowability. However, the bulk densities of TUBS-T are slightly lower, which indicates a higher porosity. It appears that the densification behavior in the present samples has a greater impact on the flow behavior. In addition to the experimentally investigated properties, the flow behavior is also influenced by the chemical composition of the particles (leading, for example, to different Van der Waals forces), which in turn determines the frictional properties and particle–particle interactions between them [107]. However, this was not considered in the present study.

Considering all curves in Figure 9, a fundamental behavior can be seen: the smaller the particle size, the worse the flow of the bulk material. This effect has been observed and discussed widely in the literature [82,108,109,110,111,112]. Furthermore, it was found that the smallest fraction <25 µm exhibits a significantly lower flowability for both materials. According to Schulze [113], dry bulk solids above 100 µm are considered non-cohesive, which is reflected in the corresponding flow curves (all values can be found in Table A2). Fine-grained particles <100 µm have a higher specific surface area, which leads to an enhancement of the existing interparticular adhesive forces (e.g., Van der Waals forces). In this size range, the adhesive forces play a greater role than the gravitational forces, which is why the latter are characterized by reduced flowability [114].

**Figure 10:**
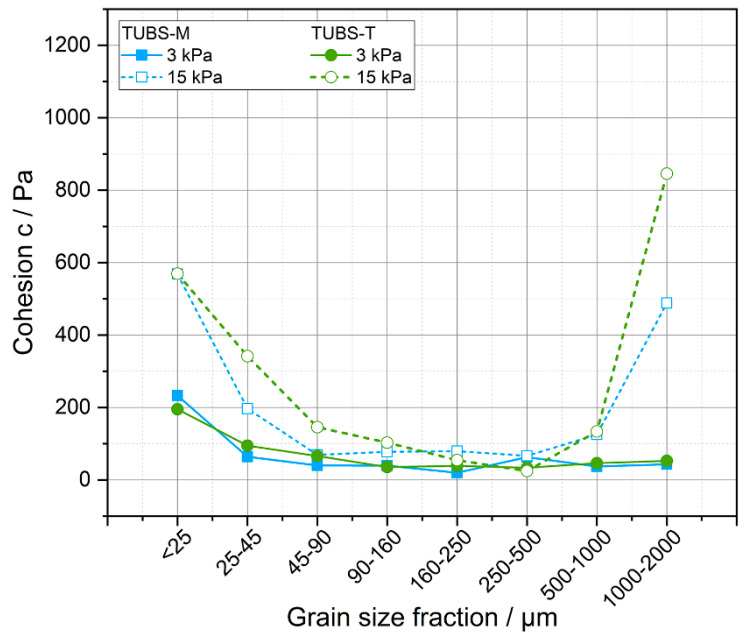
Cohesion values of the grain size fractions of TUBS-M and TUBS-T at shear stresses of 3 and 15 kPa.

For coarser particle sizes >250 µm, it is noticeable that measurement deviations result in a less linear trend of the flow curves. This is caused by the measuring principle of the ring shear tester. The shear cell consists of a bottom and a lid, the latter being equipped with lamellar carriers. Larger particles tend to get jammed in the carriers, which causes tensions that result in higher standard deviations and local stress peaks. This effect will be discussed in more detail in the next chapter (see Figure 10).

If it is not possible to perform shear tests, the Hausner ratio (*HR)* can be calculated alternatively. It is defined as the ratio of tapped *ρ_T_* to bulk density *ρ_B_* (see Table A1) and describes how sensitive a powder bed is to compaction under gravimetric forces, which also allows the description of interparticular interactions and thus, the estimation of the flowability according to Carr [115]: *HR = ρ_T_/ρ_B_*.

An *HR* of 1 means that external impacts and forces do not influence the volume of the bulk material and implies good flowability and easy powder handling. An *HR >* 1 indicates that the material compacts. The higher the value, the stronger the consolidation and the poorer the flowability. As shown in Table A1, the *HR* values for the different size fractions TUBS-M and TUBS-T range from 1.02–1.17, which indicates very good to good flowability for all samples. However, in comparison to ring shear measurements (Figure 9), the results are contradictory, because according to *HR*, the size fractions of TUBS-M show an overall more cohesive behavior. It should also be noted that the *HR* in this case only reflects the scenario at maximum compression (*N* = 500 taps). This leads to the conclusion that the *HR* is only suitable for a quick and easy estimation of the flow and solidification properties, but that more detailed measurements (e.g., ring shear tests) have to be performed to generate reliable data.

The use cases that can be described using ring shear measurements are the same as for bulk and tapped density, but flow properties describe phenomena more accurately. The complex procedure opens more room for interpretation. During the measurement, defined load conditions can be simulated, and, in particular, cohesive powders can be investigated. In addition to the applications described for densities, this method can also be used to unravel wheel damage mechanisms (e.g., Mars Exploration rovers [72]).

### 4.5. Cohesion

In the literature, cohesion is often reported for lunar regolith simulants. For the determination of this, mainly triaxial compression and (direct) shear tests are used to investigate the shear strength of lunar (simulant) soils. An overview of the methods and conditions used for some regolith simulants in the literature was provided by Monkul et al. [77]. The exact cohesion values for all investigated shear stresses for all size fractions of TUBS-M and TUBS-T are given in Table A2. The trend of the cohesion values obtained by shear tests roughly corresponds to the shape of flow functions shown in Figure 9. Cohesion values were determined from the yield loci of the flow curves.

Figure 10 shows an example for the relationship between cohesion and particle size at two different pre-consolidation stresses of 3 and 15 kPa for TUBS-M and TUBS-T samples. Here, the influence of particle–particle interactions is clearly shown, as the cohesion increases when the regolith grains become finer. Moreover, higher normal stresses result in higher cohesion values due to lower distances and more contacts between the particles. TUBS-T exhibits slightly higher cohesion values due to the morphology influence, which is particularly evident for coarser fractions, because the aspect ratios are almost identical as the particle size decreases (see Figure 6). Furthermore, it can be observed that the cohesion in general rises with increasing precompaction stress (compare Table A2). Hence, the further away from the soil surface and the deeper in the regolith bed, the more cohesive the lunar (simulant) soil behaves [97]. The high values obtained in the case of 15 kPa for the size fractions >500 µm are due to the mechanical entanglement of the particles in the carriers of the shear cell lid. This results in stress peaks that are incorrectly translated into high cohesion values by the measurement software (see Section 4.5).

In addition, actual use cases can be described on the basis of Figure 10. In the right part of the graph, especially for particles in the mm range, the just described entanglement effect can be seen. This can be taken as a possible cause for e.g., the wheel fatigue of Mars rover Curiosity [72]. These stress peaks caused by particles jamming at the wheels grousers can overstress the material locally to the point of destruction. The left area of the graph defines the lunar dust problem. As already demonstrated several times, the finest particles <25 µm show special properties. They dust equipment and adhere to most surfaces [92]. This also affects solar panels, for example, which can lead to problems in the energy supply. Cohesion values rise for particle sizes <100 µm because they get consolidated at the carriers of the shear cell lid and thus block the measuring system.

However, it must be noted that cohesion values from shear measurements represent approximate results that are determined by the extrapolation of the so-called yield loci. Cohesion values are determined at infinitely small normal stresses that cannot be measured directly. Therefore, the cohesion is always an extrapolation of the yield loci to a normal stress of zero. As a result, deviations can occur depending on the equipment and evaluation method used, and literature values should be compared with caution.

### 4.6. Geotechnical Behavior of TUBS-M, TUBS-T, and TUBS-I

**Figure 11:**
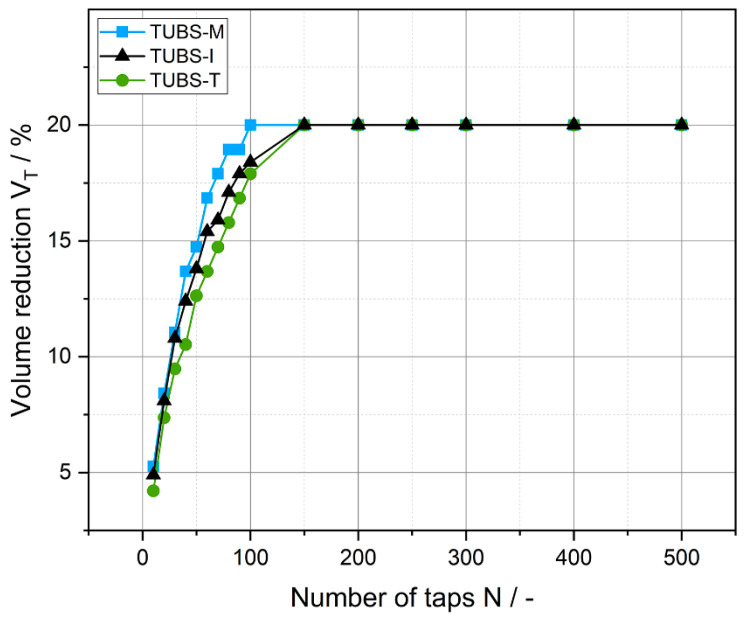
Volume reduction of the regolith simulants TUBS-M, TUBS-I, and TUBS-T as a function of the number of taps.

After properties like the flow and densification behavior of the different particle size fractions have been investigated and clarified, the geotechnical properties of the finished regolith simulants TUBS-M, TUBS-T, and TUBS-I shall now be discussed. In Figure 11, the volume reduction of the three simulants is plotted over the number of taps. All three samples reach a maximum volume reduction of 20% at about 150 taps. As already observed in Figure 7, the compaction process slows down over time until a final value is reached. The grains rearrange quickly at the beginning, and then pores and cavities are filled, predominantly with fine particles.

In Figure 8, it could be observed that size fractions >90 µm reach a maximum volume reduction of 4 to 9%. With smaller particle sizes, the percentage volume reduction increases and reaches values up to 31% for the smallest fraction. The *x*_50_ of the three simulants ranges from 76.7 to 96.0 µm (see Table 2), and thus, in terms of particle count, there are relatively few coarse grains (though high masses) in the mixture. Moreover, the high proportion of small grains (within the mixture) indicates that the finer fractions significantly influence the overall behavior of the regolith simulants [101].

**Figure 12:**
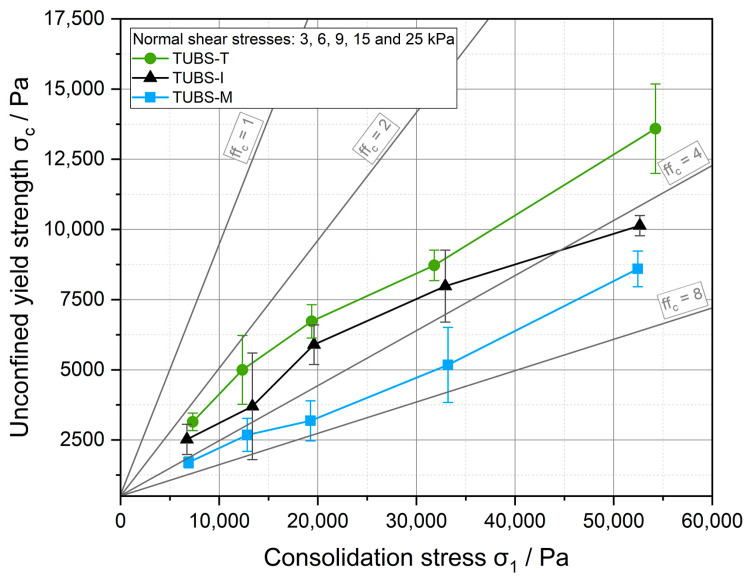
Flow behavior of the three regolith simulants at different shear stresses.

The results of the ring shear tests in Figure 12 present a pattern, which could already be predicted from the flow curves of the size fractions in Figure 9. TUBS-T exhibits a significantly higher cohesiveness than TUBS-M, among other things, due to a lower aspect ratio of the single particles. The flow curve of TUBS-I represents a logical mean value, because this simulant consists of a 50:50 mixture of TUBS-M and TUBS-T.

What is noticeable, however, are the significantly lower *ff_c_* values, which range from approx. 3 to 8 for all simulants. All separated fractions (see Figure 9), on the other hand, exhibit *ff_c_* values of around and above 10, which defines them as free-flowing. Based on the same foundation, the unconfined yield strengths *σ_c_* are up to ten times higher for the simulants with adjusted particle size distribution (PSD). Here, the interaction of different grain sizes is expressed by a broad and inhomogeneous PSD. The coarse particles are embedded in a matrix of small particles, which results in a higher bulk density (see Table A3), and thus, in a more compact material. Furthermore, the compact material shows an increased flow resistance at all investigated shear stresses. This is also a consequence of the dominant behavior of the particle–particle interactions among the fine fractions on the overall flow behavior, which is mirrored in the cohesion values (see Table A4). If the simulated soil is exposed to higher stresses, it reaches higher bulk densities and flows more poorly. In addition, the poor flowability has a significant effect on powder handling and must be taken into account when designing new equipment and devices. In particular, in areas where a lot of meteorites or dust particles impact, resulting in more fines with non-spherical morphologies, a much more cohesive behavior of the lunar soil is to be expected.

To summarize: By combining all size fractions, a much more cohesive behavior is obtained in the final simulant (see Figure 12), than for the individual fractions (see Figure 9). All three simulants exhibit a maximum compaction of about 20% (see Figure 11), while the compaction behavior of the individual fractions varies considerably (see Figure 7). Due to locally different compositions, PSDs and segregation phenomena, the behavior of the fine or coarse particles can dominate. Mission risks quickly arise, such as rovers getting stuck because the sensor technology used cannot handle all bulk-material-related scenarios.

### 4.7. Comparison of the Data with Real Lunar Regolith

Various properties of the three investigated lunar regolith simulants are compared with values of real lunar regolith in Table 2. Besides the high correlation of the chemical and mineralogical properties (see [2]), this summary shows a similar picture: The physical and geotechnical properties approach the values known from lunar regolith very accurately. It should be noted, however, that depending on the batch and measurement method, different results may occur. It can therefore be concluded that TUBS-M, TUBS-T, and TUBS-I are suitable base simulants for a variety of investigations and can be used to perform tests under realistic conditions.

## 5. Conclusions and Outlook

The literature states that there are no lunar regolith simulants that can cover the multitude of use cases needed for exploration missions and that, in most cases, no comprehensive characterization is provided along with their release [62]. To overcome this major gap, a modular system for flexible adaptable novel lunar regolith simulants was developed in earlier works [1], and detailed mineralogical and chemical analysis were published [2]. Building on this, the present study provides thorough investigations regarding the geotechnical properties of the three novel base regolith simulant systems: TUBS-M, TUBS-T, and TUBS-I (50:50 blend of TUBS-M and -T).

Individual grain size fractions ranging from a few µm up to 2 mm were investigated so observations could be better explained. TUBS-T tends to form more elongated particles than TUBS-M, with aspect ratios of 0.5–0.68 and 0.53–0.68, respectively. Microscopic and laser-scattering measurement show that fine (nano-)particles adhere to the surface of all coarser particles and can be found in all size fractions, demonstrating the huge problem with lunar dust adherence, abrasion, and penetration [91,92].

Bulk and densification measurements show that all size fractions densify quickly under little external stress. Particle sizes >100 µm reach maximum densifications of up to 10%. The maximum percentage of compaction increases with decreasing particle size and reaches over 30% in the two-digit micrometer range. With a *x*_50_ of 76.7 to 96.0 µm, the regolith simulants predominantly consist of finer particles. Ring shear measurements confirm this behavior.

Cohesion measurements further demonstrate damage mechanisms. Particles in the mm range cause local stress peaks. Cohesion values reach 820 Pa for TUBS-T and 500 Pa for TUBS-M at 15 kPa shear stress versus around 50 Pa at 3 kPa shear stress. Additionally, particles <100 µm show the same behavior (~500 Pa at 15 kPa shear stress; ~200 Pa at 3 kPa shear stress). Fine particles and lunar dust densify so quickly that the measurement system gets blocked that is directly transferable to all kinds of regolith-processing technologies.

The base simulants TUBS-M, TUBS-T, and TUBS-I, with full particle size distribution, quickly reach maximum volume reductions of 20% but show much more cohesive behavior (up to 15,000 Pa) when compared to the size fractions (up to 15,000 Pa). This shows how the strong particle–particle interactions of fine particles affect a matrix of coarse grains. All of these fluctuating properties create terrain-based challenges in the conveyance of regolith and thus affects the design of equipment.

Regarding the comparability of particle size analyses in the literature, different common particle measurement procedures were performed, showing considerable differences in the resulting data.

In summary, a novel contribution for space exploration and ISRU endeavors could be presented by further characterizing so far unique flexible adaptable lunar regolith simulants. The geotechnical investigations allow describing the observed findings not only phenomenologically but also to explain them with particle technical correlations.

Future work will address the analysis of chemically (CAS) and physically (PAS) adapted lunar regolith simulants and additive manufacturing methods, as well as the investigation of landing-pad structures and rover–regolith interaction under different load conditions. In all of these endeavors, data-driven methods (e.g., simulations and AI) and the predictability of results and scenarios play an important role.

## Figures and Tables

**Table 1:** Particle sizes of the grain size fractions of both regolith simulants.

	TUBS-M				TUBS-T			
Grain Size Fraction	*x*_10_ μm	*x*_50_ μm	*x*_90_ μm	*PDI* -	*x*_10_ μm	*x*_50_ μm	*x*_90_ μm	*PDI* -
1000–2000	1057.69	1475.19	1696.85	0.43	379	1247.82	1469.35	0.87
500–1000	578.53	856.5	1259.44	0.79	479.61	707.48	994.66	0.73
250–500	267	431	681	0.96	252	388	588	0.87
160–250	146	232	349	0.88	159	238	349	0.80
90–160	78.1	135	215	1.01	94.6	145	217	0.84
45–90	37.1	70.3	118	1.15	45.7	78	125	1.02
25–45	12.40	37.90	64.80	1.38	11.7	38.7	66.3	1.41
<25	2.28	11.30	28.60	2.33	3.12	13.6	31.6	2.09

**Table 2:** Physical properties of the investigated batch samples compared with lunar values taken from [28].

Property	TUBS-M	TUBS-I	TUBS-T	Lunar Regolith
Bulk density g cm^−3^	1.41–1.62	1.43–1.56	1.33–1.46	1.45–1.55
Tapped density g cm^−3^	1.81	1.65	1.61	na
Av. Hausner ratio -	1.19	1.10	1.15	na
Aspect ratio -	0.53–0.68	0.50–0.68	0.50–0.68	0.4–0.7 [95]
Particle size				Acc. to distribution
µm				band
*x*_10_	8.5	5.5	5.3	5–25
*x*_50_	76.7	88.6	96.0	41–130
*x*_90_	593.6	1074.9	912.3	300–2600

## Data Availability

Not applicable.

## Appendix A

**Table A1:** Bulk density, tapped density, and Hausner ratio of the grain size fractions of both regolith simulants.

	TUBS-M			TUBS-T		
Grain Size Fraction µm	Bulk Density g cm^−3^	Tapped Density g cm^−3^	Hausner Ratio -	Bulk Density g cm^−3^	Tapped Density g cm^−3^	Hausner Ratio-
1000–2000	1.27	1.49	1.17	1.25	1.34	1.08
500–1000	1.25	1.42	1.13	1.20	1.32	1.11
250–500	1.25	1.35	1.09	1.24	1.31	1.05
160–250	1.29	1.36	1.06	1.27	1.30	1.03
90–160	1.33	1.42	1.07	1.28	1.30	1.01
45–90	1.35	1.45	1.08	1.25	1.28	1.02
25–45	1.32	1.47	1.11	1.20	1.32	1.10
<25	1.20	1.32	1.10	1.10	1.15	1.04

**Table A2:** Cohesion values of the grain size fractions of TUBS-M and TUBS-T extracted from shear tests at different shear stresses.

	Cohesion/Pa									
	TUBS-M					TUBS-T				
Grain Size Fraction µm	3 kPa	6 kPa	9 kPa	15 kPa	25 kPa	3 kPa	6 kPa	9 kPa	15 kPa	25 kPa
1000–2000	43.00	93.00	98.33	488.33	507.33	52.33	135.67	131.67	845.67	997.00
500–1000	37.00	124.67	82.00	125.00	218.67	46.33	104.00	98.00	133.67	295.33
250–500	63.00	31.00	52.67	66.33	70.33	33.33	37.00	36.67	24.33	89.67
160–250	19.67	29.00	49.33	79.33	50.33	38.67	34.67	19.00	54.33	79.00
90–160	39.33	16.00	84.00	77.33	78.67	35.33	40.67	44.00	103.00	91.33
45–90	39.67	40.67	56.00	69.00	56.00	66.00	97.00	91.00	145.67	246.67
25–45	64.00	94.00	126.33	196.67	266.33	94.67	142.00	198.67	341.67	539.00
<25	232.67	318.00	412.33	568.33	877.00	195.33	332.00	438.67	569.33	848.67

**Table A3:** Cohesion values of TUBS-M, TUBS-T, and TUBS-I at different shear stresses.

	Cohesion/Pa				
Regolith Simulant	3 kPa	6 kPa	9 kPa	15 kPa	25 kPa
TUBS-M	349.67	604.67	668.67	1023.67	1902.67
TUBS-I	584.33	717.00	1300.00	1749.33	2240.67
TUBS-T	642.67	1077.67	1360.67	1714.33	2793.67

**Table A4:** Bulk densities of TUBS-M, TUBS-T, and TUBS-I at different shear stresses.

	Bulk Densities/g cm^−3^				
Regolith Simulant	3 kPa	6 kPa	9 kPa	15 kPa	25 kPa
TUBS-M	1.41	1.55	1.55	1.61	1.62
TUBS-I	1.43	1.49	1.51	1.56	1.58
TUBS-T	1.33	1.38	1.42	1.46	1.50
